# A field-study on *Leptospira *seroprevalence in dairy cows in four geographical areas in Sweden

**DOI:** 10.1186/1751-0147-53-53

**Published:** 2011-10-12

**Authors:** Elisabeth Lindahl, Sofia Boqvist, Karin Artursson, Ulf Magnusson

**Affiliations:** 1 Division of Reproduction, Department of Clinical Sciences, Swedish University of Agricultural Sciences, SE-750 07 Uppsala, Sweden; 2 Division of Bacteriology and Food Safety, Department of Biomedical Sciences and Veterinary Public Health, Swedish University of Agricultural Sciences, SE-750 07 Uppsala, Sweden; 3 National Veterinary Institute, SE-751 89 Uppsala, Sweden

**Keywords:** cow, *Leptospira*, Sweden, serology

## Abstract

**Background:**

Dairy cattle were used as sentinels for the presence of *Leptospira *infection in Swedish livestock in four regions contrasting in precipitation and temperature during the summer time. The aim of the study was to estimate the prevalence of five serovars of *Leptospira *of low pathogenicity in dairy cattle in these four regions.

**Findings:**

Around 150 blood samples were collected from five dairy farms from each region, making 610 samples in total, during December 2009. The samples were screened for *L. kirschneri *sv Grippotyphosa, *L. interrogans *sv Icterohaemorrhagiae, *L. interrogans *sv Canicola, *L. borgpetersenii *sv Sejroe and one domestic strain similar to sv Sejroe, called strain Mouse 2A using the Microscopic Agglutination Test. Six animals (1%) were seropositive for the strain Mouse 2A. Four of the positive samples were from the south-west region which also was the region with highest precipitation. There were no positive samples to any of the other serovars studied.

**Conclusions:**

The present data indicate that there is a low seroprevalence of *Leptospira *in Swedish dairy cows. These findings can be used as baseline data to investigate the effects of, for instance, climatic change or alterations in wildlife reservoir populations on the seroprevalence of *Leptospira *in the future.

## Background

The zoonosis leptospirosis, which is of worldwide distribution, is caused by different pathogenic serovars belonging to the genus *Leptospira *[1] and is endemic in most tropical and temperate climates [2]. The bacteria thrive in humid and warm climate and outbreaks of leptospirosis increase during heavy rainfall and flooding [3]. Leptospirosis is therefore of particular interest for Europe in the context of climatic change and has been recommended for specific surveillance and control measures by the World Organization for Animal Health (OIE) [4]. Similarly, the European Centre for Disease Control (ECDC) is also pinpointing leptospirosis as an important infectious disease of particular interest in Europe [5].

The severe pathogenic serovars Pomona in pigs and Hardjo in cattle have not been reported in Sweden [6]. However, in pigs reared outdoors, seroprevalences of 4 and 1% for sv Bratislava and sv Icterohaemorrhagiae have been found (Boqvist S, Eliasson-Selling L, Bergström K, Magnusson U: Association between rainfall and temperature, and seroprevalence of *Leptospira *in outdoor reared pigs, submitted). The latter study also showed that the seroprevalence for a domestic strain called Mouse 2A (similar to sv Sejroe) was 8%. In Swedish cattle, the seroprevalence of serovars with lower virulence than sv Hardjo is virtually unknown.

In the present study, dairy cattle in farms with summer grazing were used as sentinels for possible differences in seropositivity between regions with contrasting climate but similar breeds and farming practices. The aim of the study was to estimate the prevalence of five serovars of *Leptospira *of low pathogenicity in dairy cattle in four climate regions in Sweden.

## Materials and methods

**Figure 1:**
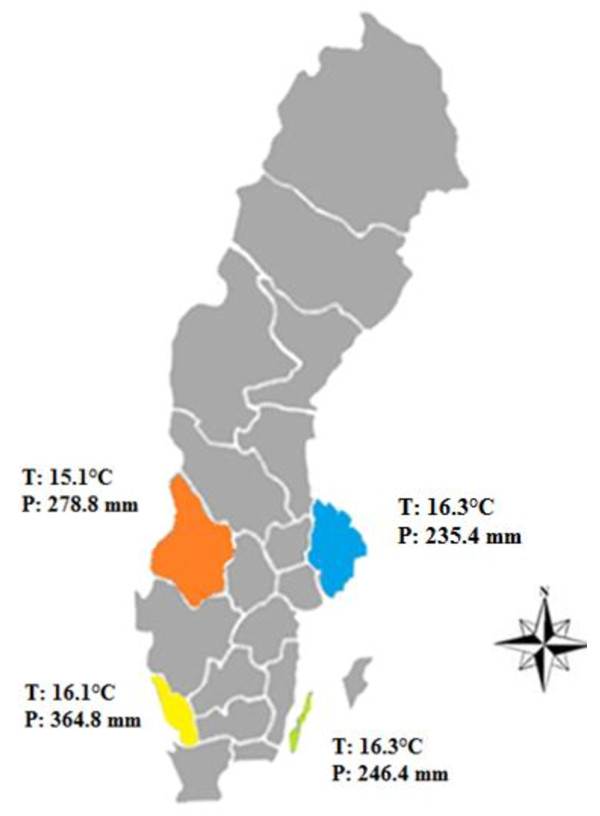
**Map showing the four regions (south west, south east, mid west and mid east) with the annual mean temperature (T) and precipitation (P) during June to August 2005-2009 where blood samples from in total 610 dairy cows were collected for *Leptospira *serology**.

The study area consisted of four different regions in Sweden contrasting in precipitation and temperature: south west (Halland's county), south east (the island of Öland), mid-west (Värmland's county) and mid-east (Uppsala and Stockholm counties) (Figure 1). From these areas, data on annual mean temperature and precipitation between June and August during the years 2005-2009 were provided by the Swedish Meteorological and Hydrological Institute (SMHI), Norrköping [7]; (Figure 1). June, July and August correspond with the grazing period for dairy cows in these regions, i.e. the period when they are most likely to be exposed to *Leptospira *in the environment.

Dairy herds of 40-60 cows were included to represent the average dairy herd size in Sweden [8]. A list of ten eligible herds with between 25 and 80 dairy cattle from each region was provided by the Swedish dairy association, Stockholm. From this list five herds per region were selected, making 20 herds in total, based on i. their geographical location, since the aim was to distribute the included herds evenly within each region; ii. the willingness of the farmers to participate in the study; and iii. the housing system, since a pre-requisite for inclusion was that the cows should be reared in a tie-stall system to facilitate the sampling.

In total, 610 serum samples were collected, ranging from 25-33 samples per herd and 150-156 per region. The median number of dairy cows per herd was 50 (ranging from 25-80) and the median number of all cattle per herd was 104 (45-180). This was sufficient to estimate a within-herd seroprevalence of around 5%, depending on herd size, with an error of 5% and a confidence interval of 95% (Win Episcope 2.0). Fisher's exact test was used to compare seroprevalences between regions. Within the herds cows were selected randomly and sampled by the same person during December 2009. Blood samples were taken from the coccygeal vein and transported in a cooler to local laboratories for centrifugation. The samples were subsequently transported frozen to the Swedish University of Agricultural Sciences in Uppsala and kept at -20°C until analysed. The study was approved by decision C 322/9 at the Ethical Committees on Animal Experiments, Uppsala, Sweden. All sera were examined by the Microscopic Agglutination Test as described previously [9] for *L. kirschneri *sv Grippotyphosa, strain Duyster, *L. interrogans *sv Icterohaemorrhagiae, strain Kantorowicz, *L. interrogans *sv Canicola, strain Hond Utrecht IV, *L. borgpetersenii *sv Sejroe, strain M84 and *L. borgpetersenii*, domestic strain Mouse 2A isolated from the kidney of a mouse caught in a Swedish pig herd (Eva Olsson Engvall, National Veterinary Institute, Uppsala, Sweden). The other serovars were selected as they are known to be of clinical importance in cattle. Notably, the well known pathogenic sv Hardjo was not included as this serovar has been surveilled regularly for in Sweden without any positive findings [10]. The cut-off value was 1:100. Of all sampled cows 65% were of the Swedish black and white breed (SLB) and 35% of the Swedish red and brown breed (SRB). The median age of all cows was 4 years (2-12). The median grazing period per year was 5 months (3.5- 6) and the dominant form of pasture was arable land (77%).

## Results and Discussion

Six (1%) of the animals were serologically positive for the strain Mouse 2A. The titers were 1:100 in five animals and 1:200 in one. Four of these animals were from three farms in the south-west region, one animal in the mid-west region and the last one in the mid-east region. There were no statistically significant differences in seroprevalence between the regions. Both breeds were represented, each with three seropositive animals. All seropositive animals were in the age interval 3-6 years.

Most studies on *Leptospira *seroprevalence in livestock are from slaughter houses without any farm data. Here farm and metrological data from 20 herds and approximately 600 cows were included. This number of animals gives an indication of the serological situation in the southern part of Sweden, even though it may be difficult to measure low titers resulting from chronic infections by the MAT [11]. Despite this difficulty, a previous study in pigs kept outdoors showed a seroprevalence of 8% against the strain Mouse 2A (Boqvist S, Eliasson-Selling L, Bergström K, Magnusson U: Association between rainfall and temperature, and seroprevalence of *Leptospira *in outdoor reared pigs, submitted). In the latter study the highest seroprevalence of *Leptospira *was found in farms in the rainier south- western region of Sweden and there was a positive association between seropositivity and rainfall. In the present study no differences in *Leptospira *seroprevalence in cattle between the regions could be demonstrated; however, it was not possible to take into account clustering of disease because of the design of the study. The present data suggest that seropositivity to pathogenic *Leptospira *in cows is low in Sweden and currently there are no reasons to believe that there are differences between regions attributable to factors such as temperature and precipitation. These findings can be used as baseline data to investigate potential future changes of *Leptospira *seroprevalence, such as climatic changes or alterations in wildlife reservoir populations.

## Competing interests

The authors declare that they have no competing interests.

## Authors' contributions

EL performed the field work, summarized the results and wrote most of the manuscript.SB planned the study, contributed to summarizing the data and writing the manuscript. KA was responsible for the MAT-analyses and commented on the manuscript. UM planned and organized the study, contributed to summarizing of data and writing the manuscript. All authors read and approved the final manuscript.
